# Peptide-Splitting Ferment of Carcinomatous Stomach

**Published:** 1913-11

**Authors:** 


PEPTIDE-SPLITTING FERMENT OF CARCINOMATOUS STOMACH. A. Graham Bryce, M. D., Pendlebury, Eng., *Med Chron.*. July, 1913. This article is an extensive review of literature, checked by personal investigation. Preformed tryptophan in the stomach contents (Erdmann-Winternitz test) is rather characteristic of cancer but Neuerbauer and Fischer regard it as due to regurgitation from the intestine. It is not at present regarded as of great diagnostic value and was not so stated by its discoverers. Neuerbauer and Fischer's modification of the test by letting the gastric contents act upon glycyl-tryptophan is regarded as more definitely diagnostic, but a positive reaction has been obtained in only 67.7 per cent. of demonstrated cases of cancer and some of these have not been positive except after repeated trials. (Note. It should be remembered that a reaction in 50 per cent. of cases has a zero value.) There are also interferences to be considered. Many bacteria, including the colon and typhoid bacillus and B. proteus, split peptides, although by filtering the matter investigated and adding toluol, this action can be largely eliminated. Free HCI to the amount of 90 degrees decinormal inhibits the action of the peptide-splitting ferment of carcinoma, but this degree of acidity is rarely found and practically never in cancer cases. Trypsin, regurgitated from the intestine (or formed in the stomach?) as well as blood may also cause a peptide splitting.

(Note. While in an academic sense and as indicating potential practical developments, tests that are merely characteristic of a given condition have a considerable value, for practical diagnostic purposes, it is better not to use such a test at all, unless both patient and physician can maintain an absolutely judicial attitude. Unfortunately, all kinds of laboratory tests are given a degree of reliance superior to that given ordinary clinical diagnostic methods, and, often, without the warrant of facts.)

